# Effective Monotherapy with Amrubicin for a Refractory Extrapulmonary Small-Cell Carcinoma of the Liver

**DOI:** 10.1155/2009/538081

**Published:** 2009-06-16

**Authors:** Taichi Isobe, Shunichi Yanai, Hitoshi Kusaba, Shinichiro Yada, Yosuke Kuroda, Sadafumi Tamiya, Takayuki Matsumoto, Eishi Baba, Mine Harada

**Affiliations:** ^1^Department of Medicine and Biosystemic Science, Graduate School of Medical Sciences, Kyushu University, Maidashi 3-1-1, Higashi-Ku, Fukuoka 812-8582, Japan; ^2^Department of Medicine and Clinical Science, Graduate School of Medical Sciences, Kyushu University, Maidashi 3-1-1, Higashi-Ku, Fukuoka 812-8582, Japan; ^3^Department of Anatomic Pathology, Graduate School of Medical Sciences, Kyushu University, Maidashi 3-1-1, Higashi-Ku, Fukuoka 812-8582, Japan

## Abstract

Small-cell carcinoma of the liver is a rare neoplasm, and no standard treatment for it has yet been established. A 72-year-old man with an extensive disease stage of small-cell carcinoma of the liver was treated with systemic chemotherapy consisting of cisplatin and etoposide (PE) followed by irinotecan. Although the masses were markedly decreased once after the sixth course of PE, amrubicin monotherapy as third-line chemotherapy was started because the hepatic masses had increased again. The administration of amrubicin was repeated in 8 courses with regression of the disease, resulting in a 26-month survival since the first-line chemotherapy was started. This is the first case report of a refractory EPSCC successfully treated with amrubicin.

## 1. Introduction

Extrapulmonary small-cell carcinomas (EPSCCs) are found in 2.5% to 4.0% of all small-cell carcinoma cases [1–3]. The common primary sites of EPSCCs are the salivary gland, esophagus, stomach, pancreas, colon, and cervix [4]. The prognosis for patients with an advanced small-cell carcinoma is poor, because of the aggressive phenotype and the high frequency of metastasis. A standard treatment for EPSCCs has not yet been established. Some cases have been treated with a chemotherapeutic regimen for small-cell lung carcinoma (SCLC), such as cisplatin (CDDP) plus etoposide or CDDP plus irinotecan [1–4]. Recently, amrubicin, which is a totally synthetic 9-aminoanthracycline and a potent DNA topoisomerase II inhibitor, has been demonstrated to be effective for previously treated SCLCs [5, 6]. We herein report a patient with advanced small-cell carcinoma of the liver, who was successfully treated with amrubicin monotherapy as third-line chemotherapy following cisplatin, etoposide, and irinotecan, thus resulting in a long-term survival.

## 2. Case Report

**Figure 1:**
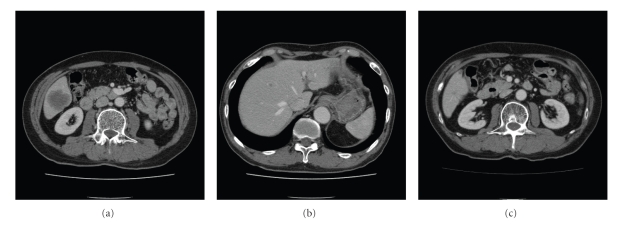
Abdominal computed tomography. Computed tomography revealed longest diameter 40 mm-sized hepatic mass in the posterior inferior segment of the liver (a) and small nodules scattered throughout both hepatic lobes (b). After 6 courses administration of amrubicin as a third-line chemotherapy, the mass decreased (c).

**Figure 2:**
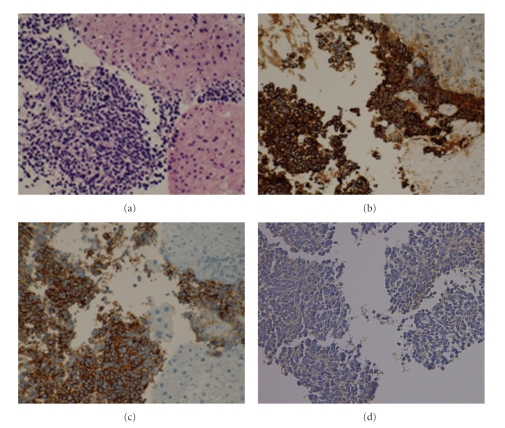
Immunohistochemical examination of small-cell carcinoma of the liver. (a) Tumor cells display large hyperchromatic nuclei and scanty cytoplasm, H&E. (b) Immunohistochemically, the atypical cells are positive for AE1/AE3, (c) N-CAM and (d) NSE. Magnification ratio: 200 times.

A 72-year-old man was referred to us in May 2005, for further examination of a gastric polyp. On physical examination, his heart rate was 60/min and his blood pressure was 132/78 mmHg. The abdominal examination was normal. According to the Eastern Cooperative Oncology Group (ECOG) criteria, the patient showed a performance status of 0. The results of the routine laboratory tests were as follows: total bilirubin, 0.6 mg/dL; AST, 16 IU/L; ALT, 13 IU/L; hepatitis B viral surface antigen and hepatitis C viral antibody were negative. The serum level of *α*-Fetoprotein was normal. The serum levels of neuron-specific enolase (NSE), gastrin-releasing peptide precursor (ProGRP), and carcinoembryonic antigen (CEA) were elevated at 13.3 ng/mL (normal range; ~10 ng/mL), 408.0 pg/mL (normal range; ~46.0 pg/mL), and 74.8 ng/mL (normal range; ~3.2 ng/mL), respectively. A chest radiograph showed clear lung fields. An ultrasonographic examination revealed a hypoechoic mass in the posterior inferior segment of the liver. Computed tomography (CT) scans of the abdomen revealed that the hepatic mass of the longest diameter (40 mm) in size was seen in the posterior inferior segment of the liver and that small nodules were scattered throughout both of the hepatic lobes (Figures 1(a) and 1(b)). A needle biopsy specimen obtained from the hepatic mass demonstrated some nests of atypical cells having large hyperchromatic nuclei and scanty cytoplasm. Immunohistochemically, the atypical cells were positive for cytokeratin (AE1/AE3 and CAM5.2) and neuroendocrine markers (N-CAM and NSE), but negative for a lymphocytic marker (LCA), which was consistent with the features of a small-cell carcinoma (Figure 2). Additionally, CK-19, CK-20, and hepatocyte specific antigen were negative. Esophago-gastroduodenoscopy (EGD) revealed a type IIc gastric cancer in the posterior wall of the lower body of the stomach. A chest X-ray, CT scan of the chest, bronchoscopy, and cytological examination of the sputum showed no evidence of primary lung cancer. CT scans of the brain and a scintigram of the bone revealed no metastatic lesions. The final diagnosis was an extensive disease stage of small-cell carcinoma of the liver, and an early gastric cancer.

The patient was initially treated with systemic chemotherapy consisting of both CDDP and etoposide. One hundred mg/m^2^ of etoposide were infused on days 1, 2, and 3. CDDP was infused at a dose of 80 mg/m^2^ over 2 hours, with adequate hydration, on day 1. G-CSF was administered from day 5 until neutrophil recovery. This regimen was repeated every 4 weeks, and the patient received 6 courses of the chemotherapy. Toxicity was graded according to the Common Terminology Criteria for Adverse Events (CTC-AE), version 3. In the first course, both grade 2 anorexia and grade 1 increase in serum creatinine were observed. Next, the dose of CDDP was reduced to 64 mg/m^2^. Grade 2 anorexia, grade 4 neutropenia, and grade 2 anemia were observed in all of the courses. After the sixth course, grade 1 neuropathy was observed. The response of the measurable lesions was assessed by RECIST criteria [7]. CT scans thereafter revealed a reduction of the hepatic mass in the posterior inferior segment, from 40 mm to 25 mm after the second course, and further to 10 mm after the sixth course, thus indicating a partial response. The hepatic small nodules also disappeared. The serum levels of NSE and ProGRP decreased to 1.9 ng/mL and 37.3 pg/mL, respectively. Next, the early gastric adenocarcinoma, which did not respond to the chemotherapy, was completely removed by an endoscopic mucosal resection (EMR). The patient was free from progression of the tumor for 8 months. After regrowth of the hepatic masses, a biweekly administration of 150 mg/m^2^ of irinotecan was performed. The hepatic mass, however, increased in size by 27 mm and a right pulmonary metastatic lesion in segment 8 newly appeared after the fifth course of irinotecan. The serum ProGRP was also increased, up to 593 pg/mL. The administration of amrubicin as a third-line chemotherapy was then started in August 2006. Forty mg/m^2^ of amrubicin were infused on days 1, 2, and 3 every 4 weeks. Grade 1 anorexia, grade 4 neutropenia, and grade 2 anemia were observed in all of the courses. G-CSF had not been used, because the duration of the grade 4 neutropenia was only a few days without febrile neutropenia. CT scans thereafter revealed a reduction of the hepatic mass in the posterior inferior segment, from 27 mm to 15 mm after the second course and further to 9 mm after the sixth course, and the other hepatic and pulmonary nodules were also reduced in size, indicating a partial response (Figure 1(c)). In addition, the serum ProGRP was decreased to 166.1 pg/mL. The administration of amrubicin has been repeated in 8 courses so far. The patient died of progression of cancer 14 months after the initiation of amrubicin monotherapy, and 26 months after the initiation of the first-line chemotherapy.

## 3. Discussion

In the literature, there are only 7 cases with a primary small-cell carcinoma of the liver [8–11]. In these reports, 5 of the 7 patients were treated with a resection or chemotherapy. One of them survived for 67 months after a right lobectomy [8]. However, 3 of the patients that were treated with a partial hepatectomy and/or systemic chemotherapy survived for 5, 13, and 15 months, respectively, [9–11].

According to previous reports, the patients with EPSCCs, especially in the cases of limited-disease treated by local modalities, have slightly better overall survival than those with SCLCs [12–14]. Although the pathological mechanism of the superiority of EPSCCs to SCLCs has not yet been clarified, several biological features have been reported. The chromosomal deletion of 3p has been described in SCLCs, but not in EPSCCs [15]. In addition, while the overexpression of Bcl-2, an antiapoptotic protein, has been detected in 75% to 95% of SCLC specimens, only 33% of gastrointestinal small-cell carcinoma specimens show the Bcl-2 overexpression [14, 16]. The origins of EPSCCs were not known well. In this case, it was not clarified whether the cancer originated from hepatocyte or bile duct cell, because immunohistochemical examinations revealed that none of CK-19, CK-20, and hepatocyte specific marker was positive as previously reported [11]. Although Zanconati et al. reported that CK-18 and CK-19 were positive in their cases, it is not clear whether the difference in immunohistochemical examinations predicts efficacy of amrubicin [9].

Systemic chemotherapy, however, has been reported to be equally effective regardless of the location of the primary site, even though a standard regimen for this disease has not yet been established. The current standard chemotherapy for extensive-disease SCLC is a combination of CDDP and etoposide or irinotecan. The combination of CDDP with etoposide or irinotecan produced good effects; the response rates were 68% or 84% and the median survival times (MST) were 9.4 months or 12.8 months, respectively, [17]. Furthermore, amrubicin, a totally synthetic 9-aminoanthracycline, has recently emerged as a new candidate agent for the treatment of SCLCs. Amrubicin is converted to amrubicinol in human bodies, and amrubicinol mainly inhibits DNA topoisomerase II [6]. Monotherapy with amrubicin for refractory or relapsed SCLCs had a response rate of 52% with an MST of 11.2 months [5]. Combination therapy with CDDP plus amrubicin for previously untreated SCLSs has demonstrated a high response rate of 88% and an MST of 13.6 months [18]. In our report, the patient with advanced small-cell carcinoma of the liver was treated with etoposide, DNA topoisomerase II inhibitor, and irinotecan, DNA topoisomerase I inhibitor, previously. He then administered amrubicin as a third-line chemotherapy. After 2 courses of monotherapy with amrubicin, the hepatic masses showed a marked regression, resulting in a partial response. The patient has been doing well for 14 months since the third-line chemotherapy was started. This case suggested that (1) amrubicin was a potent DNA topoisomerase inhibitor or (2) amrubicin had other mechanisms in the antitumor avtivity. To our knowledge, this is the first case report of a refractory EPSCC successfully treated with amrubicin. Although the prognosis of an EPSCC is extremely poor due to the highly aggressive behavior, effective second- and third-line treatments are important in improving the prognosis. Therefore, in light of this, a clinical trial of amrubicin for EPSCC treatment is warranted.
